# Two decades of global research on tetanus vaccines and tetanus immunoglobulins: a comprehensive bibliometric analysis and integrative review (2000–2025)

**DOI:** 10.3389/fcimb.2026.1766140

**Published:** 2026-03-13

**Authors:** Chaoxi Zhou, Yongbo Zuo, Jianhua Ma, Zhilin Liang, Chuanyi Zang

**Affiliations:** 1 Department of Emergency Surgery, Beijing Geriatric Hospital, Beijing, China; 2 Department of Orthopedics, Beijing Geriatric Hospital, Beijing, China; 3 Department of Emergency Surgery, Beijing HaiDian Hospital, Beijing, China

**Keywords:** bibliometric analysis, conjugate vaccine, maternal immunization, monoclonal antibodies, tetanus immunoglobulin, tetanus toxoid

## Abstract

**Background:**

Tetanus is a severe but vaccine-preventable disease controlled by tetanus toxoid (TT) immunization and tetanus immunoglobulin (TIG) therapy. Although maternal–neonatal tetanus has been eliminated in many regions, coverage gaps and sporadic cases persist in low-resource settings. As a longstanding component of global immunization and a key conjugate carrier platform, tetanus research remains essential despite advances in novel vaccine technologies. However, the global research landscape on tetanus vaccines and immunoglobulins remains unmapped.

**Objective:**

This study aimed to conduct a comprehensive bibliometric analysis of publications on TT and TIG from 2000 to 2025, to characterize research dynamics, leading contributors, and thematic evolution.

**Methods:**

We retrieved publications from the Web of Science Core Collection (SCI-Expanded) for 2000–2025 using relevant keywords (“tetanus vaccine*” OR “tetanus toxoid*” OR “tetanus immunoglobulin*” OR “tetanus immune globulin*” OR “tetanus antitoxin*”). Following screening, 2,949 English-language articles and reviews were included. Bibliometric analyses (publication trends, funding agencies, co-authorship, co-citation, and keyword co-occurrence) were performed using VOSviewer, CiteSpace, and the bibliometrix R package.

**Results:**

A total of 2,949 publications were analyzed. Annual output remained stable (mean ≈ 113.4), with citations peaking in 2021 (n=7,291). The USA led in volume (1,130 papers), while Switzerland achieved the highest impact (88.41 citations/article). The Centers for Disease Control and Prevention (CDC) and University of Oxford emerged as central hubs, with GlaxoSmithKline (GSK) illustrating the role of industry-academic partnerships. A strategic funding shift occurred around 2010: while the National Institutes of Health (NIH) drives foundational research, the Bill & Melinda Gates Foundation (BMGF) has become the engine for translational equity. Evolutionary trends show a shift from early cellular mechanisms toward maternal immunization coverage, implementation determinants in low- and middle-income countries (LMICs), and the expanding utility of tetanus toxoid (TT) as a conjugate vaccine carrier.

**Conclusion:**

Tetanus research has evolved into a dynamic model for modern vaccinology, bridging classical immunization and next-generation technology. Beyond maternal protection, the field is expanding toward conjugate platforms and recombinant biologics. Achieving global elimination requires aligning these biotechnological innovations with equitable implementation strategies in low-resource settings.

## 1 Introduction

Tetanus remains a severe but preventable infectious disease caused by *Clostridium tetani*, characterized by painful muscle spasms and high mortality, particularly in low-resource settings (Yen and Thwaites, 2019; Li et al., 2023). Despite the remarkable global progress following the introduction of tetanus toxoid (TT) vaccines and tetanus immunoglobulins (TIG), the disease continues to pose a public health challenge in many developing regions (Lambo and Nagulesapillai, 2012; Uleanya, 2018; Yusuf et al., 2021). Recent global analyses indicate that although most countries have achieved maternal and neonatal tetanus elimination, sporadic cases and coverage gaps persist, particularly in low- and middle-income regions with limited immunization access and health infrastructure (Johns et al., 2023).

Over the years, extensive scientific efforts have been devoted to improving vaccine formulations, delivery strategies, and immunological understanding of TT-based vaccines (Zaman et al., 2013; Ghalavand et al., 2018; Oldrini et al., 2023). In parallel, research on TIG and antitoxin therapy has advanced, supporting both prophylactic and post-exposure management (Tosun et al., 2017; Loan et al., 2018). However, the global research landscape on tetanus vaccines and immunoglobulins has not been comprehensively mapped, and the evolution of collaborative networks, influential institutions, and emerging research themes remains underexplored.

Bibliometric analysis provides a quantitative approach to summarize and visualize scientific progress by examining publication outputs, citation trends, and collaboration patterns (Zhou et al., 2025). Such analyses have been successfully applied in diverse vaccine-related fields, including COVID-19 (Ahmad et al., 2021b), influenza (Liu et al., 2018), and HPV vaccination (Deng et al., 2023), revealing hotspots and knowledge structures that guide future research priorities. Nevertheless, to our knowledge, no bibliometric study has systematically examined the global research trends, thematic evolution, and knowledge structures surrounding tetanus vaccines and immunoglobulins. Given the maturity of the tetanus field compared to rapidly expanding domains like COVID-19 or HPV vaccination, a comparative perspective is essential to understand how this “traditional” research area adapts to modern immunological challenges and continues to serve as a fundamental pillar in the wider landscape of vaccinology.

Therefore, this study aims to conduct a comprehensive bibliometric analysis of global publications on tetanus vaccines and immunoglobulins from 2000 to 2025, based on data retrieved from the Web of Science Core Collection (WoSCC). By analyzing publication trends, leading authors, institutions, countries, co-citation networks, and keyword co-occurrence, this work seeks to provide a macroscopic overview of research dynamics in this field, identify emerging directions, and inform future strategies for tetanus prevention and immunization research.

## 2 Materials and methods

### 2.1 Literature search and data collection

This bibliometric study aimed to characterize the research landscape surrounding TT and TIG. Relevant publications were retrieved from the Web of Science Core Collection (WoSCC), specifically using the Science Citation Index Expanded (SCI-EXPANDED) database, known for its comprehensive coverage and rigorous indexing standards. The search strategy employed the query: TS=(“tetanus vaccine*” OR “tetanus toxoid*” OR “tetanus immunoglobulin*” OR “tetanus immune globulin*” OR “tetanus antitoxin*”), with the publication period spanning from January 1, 2000, to July 30, 2025. Although 2025 data are incomplete (covering only the first seven months), they were intentionally included to provide the most up-to-date overview of research trends at the time of analysis.

**Figure 1:**
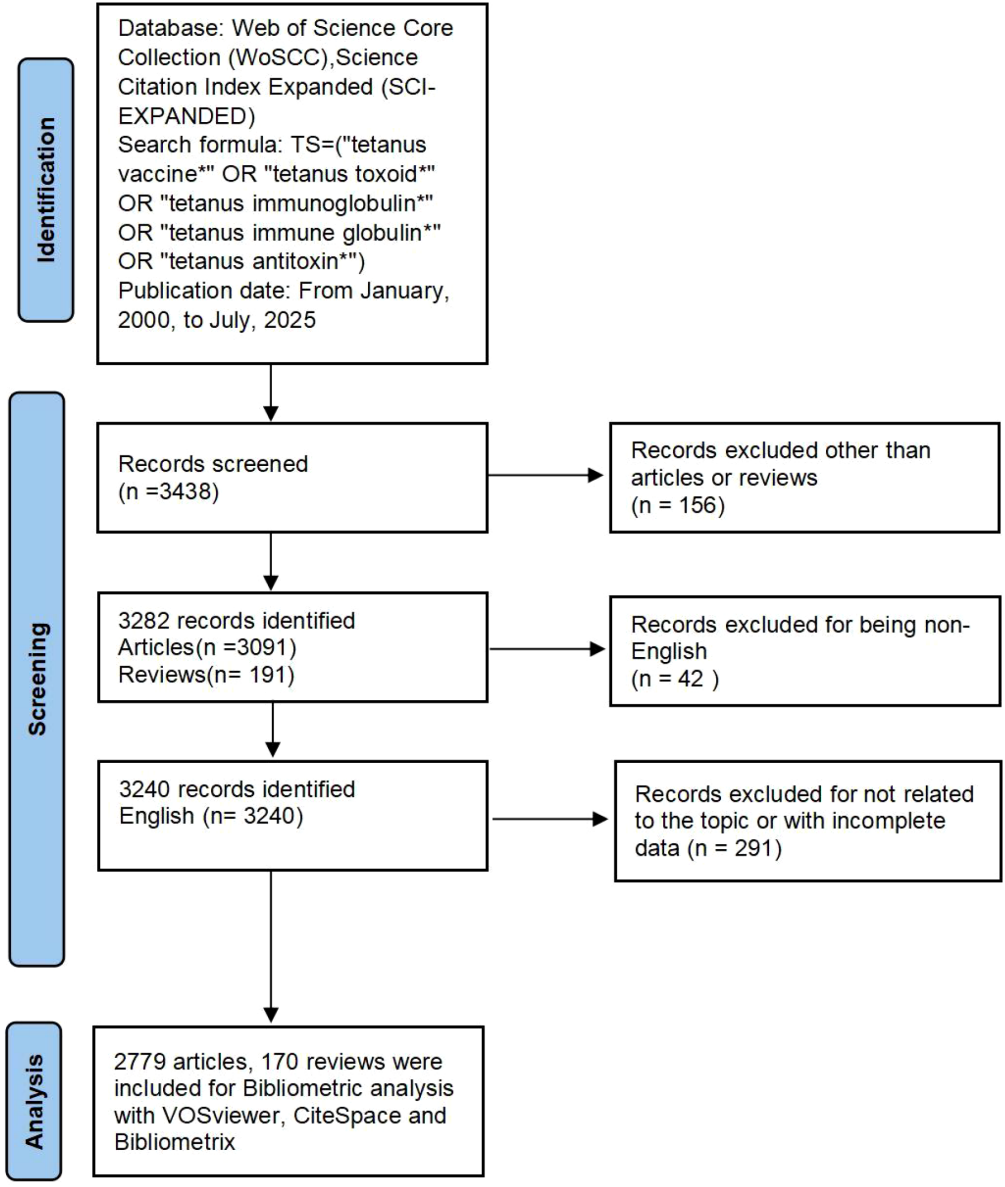
Flowchart of the literature selection and screening process. The diagram illustrates the step-by-step retrieval from the Web of Science Core Collection (WoSCC), the manual screening criteria used to ensure clinical relevance, and the final dataset of 2,949 documents analyzed.

To ensure consistency and avoid discrepancies caused by database updates, the entire literature search was performed on a single day—August 12, 2025. Three reviewers independently screened all retrieved records according to predefined inclusion and exclusion criteria. Only peer-reviewed articles and reviews published in English and focusing on TT or TIG were included. Records were excluded if they were irrelevant to the research topic, contained incomplete data, or primarily focused on veterinary studies. This manual filtration was crucial to maintain a focused medical perspective, as a substantial portion of the initial search results pertained to veterinary medicine, which would have distorted the bibliometric indicators for human medical research. After rigorous screening, a total of 2,949 publications were retained for bibliometric analysis, comprising 2,779 original articles and 170 reviews. The final dataset was exported in plain text format for subsequent analysis (**Figure 1**).

In order to assess whether WoSCC sufficiently represents the literature landscape for this research topic, and following the feasibility-check approach adopted by Liu et al (Liu et al., 2025), we additionally conducted a brief search in PubMed using a comparable set of keywords and inclusion criteria. This search identified a total of 3,050 relevant records, whereas WoSCC retrieved 2,949 documents (see **Supplementary Material 1** for details). The limited documents volume change supports the idea that WoSCC already captures the majority part of relevant publications on tetanus vaccines and immunoglobulins. Furthermore, WoSCC (SCI-EXPANDED) was selected for its high-quality citation data and consistent metadata, which are indispensable for complex bibliometric mapping. Consequently, this database offers sufficient breadth to capture the core literature, enabling a dependable analysis of major research trends and conceptual developments.

### 2.2 Bibliometric analysis

To comprehensively examine the selected literature on this area, multiple bibliometric tools were employed to explore patterns in institutional affiliations, thematic evolution, and international research collaboration. VOSviewer (version 1.6.20) was utilized to generate visual representations of institutional contributions, keyword co-occurrence, and thematic density maps. To map the landscape of global scientific collaboration, Scimago Graphica was applied, offering intuitive depictions of cross-national partnerships. CiteSpace (version 6.2.R3) was utilized to identify citation bursts and generate dual-map overlays, thereby revealing emerging research frontiers and disciplinary linkages. Additionally, we utilized CiteSpace to perform a systematic analysis of funding agencies to identify the primary drivers of research investment.

Furthermore, the bibliometrix R package was used to generate a Three Fields Plot, mapping the connections among countries/regions, journals, and institutions, thereby illustrating the structural relationships and knowledge flow across these domains. In all visual analyses, nodes represent distinct bibliometric units-such as country/region, institutions, or thems-with node size reflecting relative prominence or frequency. Color coding and edge thickness were used to convey clustering patterns, chronological progression, and the strength of collaborative ties.

## 3 Results

### 3.1 Annual publication and citation trends

**Figure 2:**
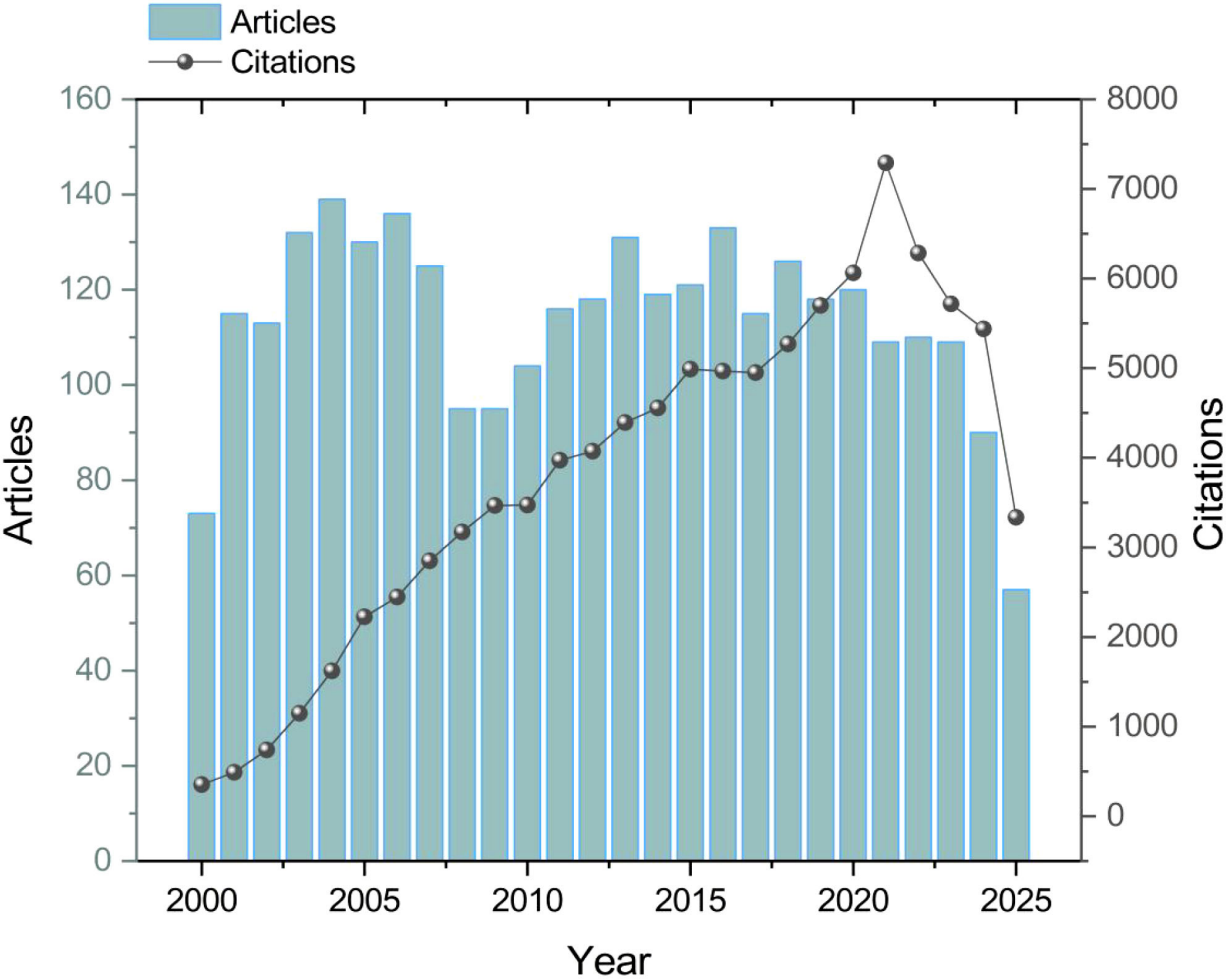
Annual trends of publication output and citation counts (2000–2025). The bar chart represents the number of annual publications, while the line graph indicates the cumulative annual citations, reflecting the growing academic impact of tetanus vaccine research.

**Figure 2** presents the annual trends in publications and citations related to TT and TIG from 2000 to 2025. The number of publications exhibited moderate fluctuations over the study period, ranging from 73 in 2000 to a maximum of 139 in 2004 (mean = 113.4 per year). Publication output increased from 73 in 2000 to 139 in 2004, followed by a slight decline and subsequent stabilization at approximately 100–130 publications annually between 2005 and 2023, before decreasing to 90 in 2024.

Annual citation counts showed a more pronounced upward trajectory, rising from 353 in 2000 to a peak of 7,291 in 2021. Citation growth was particularly marked in the early 2000s, with counts increasing from 353 in 2000 to 2,224 in 2005, and continuing to rise throughout the 2010s, reaching 4,988 in 2015 and 6,063 in 2020. Following the 2021 peak (n = 7,291), citation counts decreased to 6,283 in 2022, 5,718 in 2023, and 5,439 in 2024. The 2025 data, comprising 57 publications and 3,336 citations, reflect records available up to July 31, 2025, and therefore represent an incomplete year. Consequently, the observed decrease in 2025 counts does not indicate a decline in field activity but is a result of the truncated data collection period.

### 3.2 Geographical distribution and international collaboration

**Table 1** summarizes the top 10 productive countries. While the USA, England, and Germany dominated in total publication volume, a discrepancy between quantity and scientific impact was observed. For instance, Switzerland achieved the highest average citations (88.41), significantly surpassing the USA (36.84), suggesting a high concentration of influential, high-quality research in Swiss institutions. Additionally, the rise of India (187 publications) reflects the shifting focus toward tetanus-endemic regions, where domestic research capacity is rapidly expanding to meet local public health needs.

**Table 1:** Top 10 countries/regions contributing to TT and TIG research.

Rank	Country/region	Publications	Citations	Citation per article
1.00	USA	1130	41626	36.84
2.00	England	350	14109	40.31
3.00	Germany	214	9170	42.85
4.00	India	187	4767	25.49
5.00	Belgium	171	5103	29.84
6.00	Netherlands	152	5482	36.07
7.00	France	150	5238	34.92
8.00	Switzerland	140	12377	88.41
9.00	Australia	138	4682	33.93
10.00	Canada	130	4711	36.24

**Figure 3:**
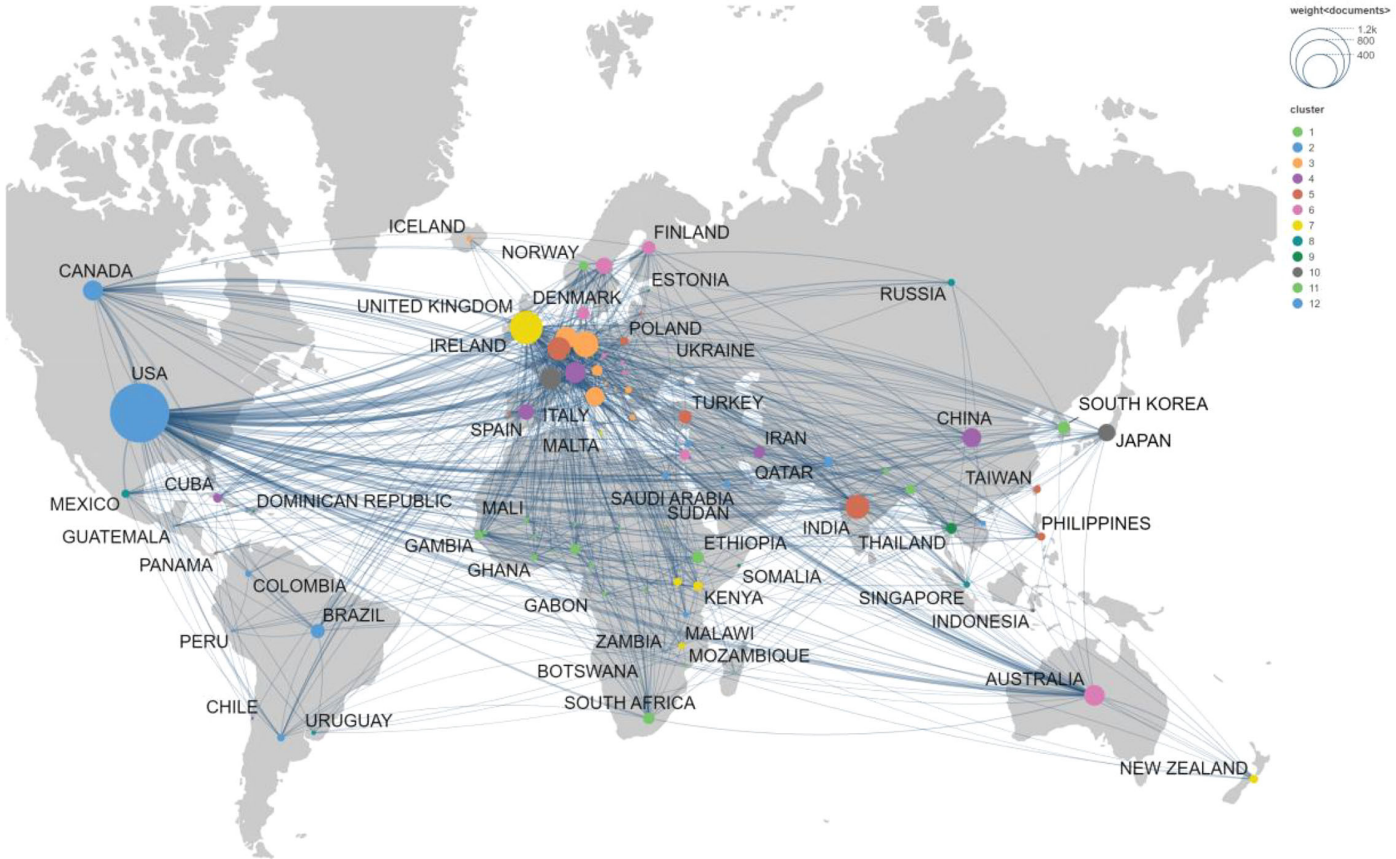
Global distribution and international collaboration network. The world map shows the geographic distribution of research output combined with a network visualization of co-authorship ties. Node size represents the number of publications, while the thickness and number of lines between nodes represent the strength of collaboration between different countries/regions.

**Figure 3** presents a world map visualization of international collaborations in research on TT and TIG, based on VOSviewer co-authorship network analysis, with 12 identified clusters. The USA emerged as the central hub, exhibiting the highest number of links, total link strength, and publications, underscoring its pivotal role in global research coordination. Strong interconnections were observed within European countries, including UK, France, Germany, Italy, and the Netherlands, extending to North American and Asian nodes. A prominent group of African nations, such as Ethiopia, Kenya, and South Africa, showed notable intra-continental ties and connections to international partners, reflecting collaborative efforts in endemic regions. Asian countries like India, China, and Japan formed key nodes with growing link strengths, indicating increasing research integration. Overall, these patterns illustrate a collaborative landscape dominated by high-income countries/regions but with significant involvement from low- and middle-income areas, particularly in regions addressing regional health challenges.

### 3.3 Institutional contributions and collaboration network

**Table 2** identifies the most influential institutions. The prominence of the Centers for Disease Control and Prevention (CDC) and the University of Oxford underscores the leadership of national public health agencies and premier academic centers in driving global tetanus policy and vaccine trials. Notably, GlaxoSmithKline (GSK) emerged as the only corporate entity in the top five, highlighting the critical role of industry-academic partnerships in the development and large-scale manufacturing of tetanus toxoids.

**Table 2:** Top publishing institutions in the field of TT and TIG.

Rank	Organization	Country/Region	Publications	RCitations	Citation per article
1	Centers for Disease Control and Prevention	USA	124	5102	41.15
2	University of Oxford	UK	65	2154	33.14
3	Harvard University	USA	64	3029	47.33
4	GlaxoSmithKline Biologicals	Belgium	58	2064	35.59
5	University of Washington	USA	49	2977	60.76
6	University of Maryland	USA	43	1220	28.37
7	London School of Hygiene & Tropical Medicine	UK	42	2574	61.29
8	World Health Organization	Switzerland	42	1905	45.36
9	Sanofi Pasteur	France	38	703	18.50
10	University of California, Los Angeles	USA	37	1594	43.08

**Figure 4:**
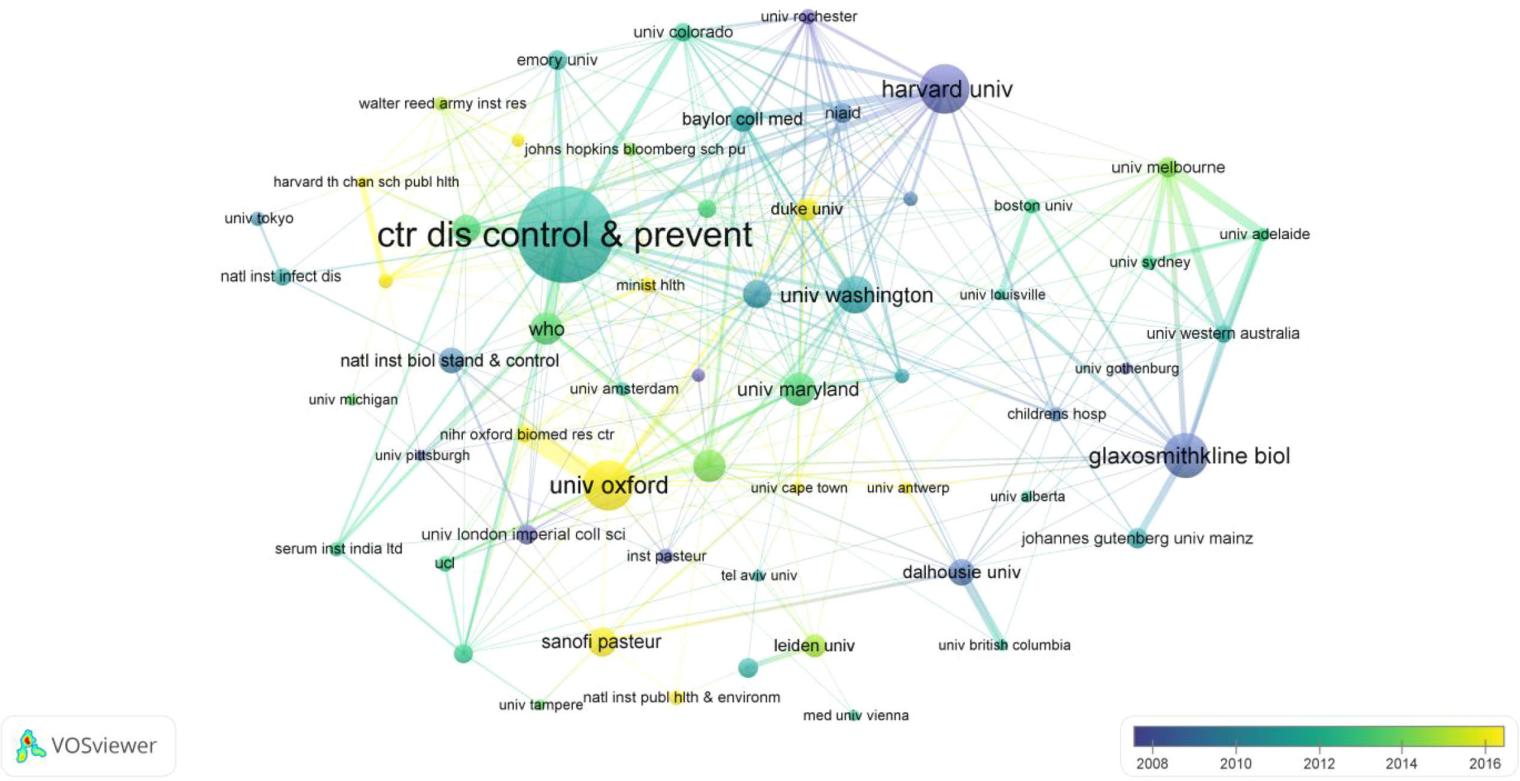
Inter-institutional collaboration network. The visualization displays partnerships between leading organizations. Node size reflects publication volume. The thickness and number of lines between nodes represent the strength of collaboration between different institutions. The color gradient indicates the average publication year, shifting from blue (earlier) to yellow (recent).

**Figure 4** depicts institutional collaboration networks via VOSviewer, overlaid with average publication years (blue for earlier periods around 2008, yellow for later ones around 2016). The CDC appeared as the most central institution, with extensive collaborative links to multiple research organizations worldwide. Other prominent institutions included the University of Oxford, Harvard University, the University of Washington, the University of Maryland, and the World Health Organization (WHO), all of which demonstrated strong connectivity within the network. Recent activity was apparent in yellow nodes like the University of Oxford, Sanofi Pasteur, and Duke University, indicating sustained research momentum into later years. In contrast, earlier contributions clustered around blue nodes such as the Harvard University, Dalhousie University and GSK Biologicals.

### 3.4 Analysis of funding agencies

The funding landscape (**Table 3**) reveals a significant geographic concentration and a strategic temporal evolution of research focus. Major investment is primarily driven by U.S. and UK organizations, notably the National Institutes of Health (NIH) and its specialized institutes—including the National Institute of Allergy and Infectious Diseases (NIAID) and the National Cancer Institute (NCI). The recurrence of NIH-affiliated entries across different years (e.g., 2000, 2008, and 2009) underscores its sustained funding cycles for foundational vaccinology. A pivotal shift occurred around 2010 with the emergence of the Bill & Melinda Gates Foundation (BMGF). By prioritizing global health equity, BMGF facilitated a transition from government-led basic research toward philanthropic initiatives specifically targeting tetanus elimination in low- and middle-income countries (LMICs). Furthermore, the involvement of diverse bodies like the National Institute of Diabetes and Digestive and Kidney Diseases (NIDDK) highlights the multidisciplinary utility of tetanus-related agents as molecular tools in broader biomedical research.

**Table 3:** Temporal distribution and ranking of major funding agencies in the field of TT and TIG.

Rank	Funding agency full name	Count	Year	Country/region
1	National Institute of Allergy and Infectious Diseases, National Institutes of Health	80	2000	USA
2	Medical Research Council	67	2005	UK
3	Bill and Melinda Gates Foundation	65	2010	USA
4	National Institutes of Health	35	2008	USA
5	Wellcome Trust	28	2005	UK
6	National Cancer Institute, National Institutes of Health	27	2000	USA
7	National Institutes of Health	26	2009	USA
8	National Center for Research Resources, National Institutes of Health	23	2000	USA
9	National Natural Science Foundation of China	18	2004	China
10	National Institute of Diabetes and Digestive and Kidney Diseases, National Institutes of Health	14	2000	USA
10	National Institute of Allergy and Infectious Diseases	14	2001	USA
10	Public Health Service, Department of Health and Human Services	14	2000	USA

### 3.5 Journal analysis

**Figure 5:**
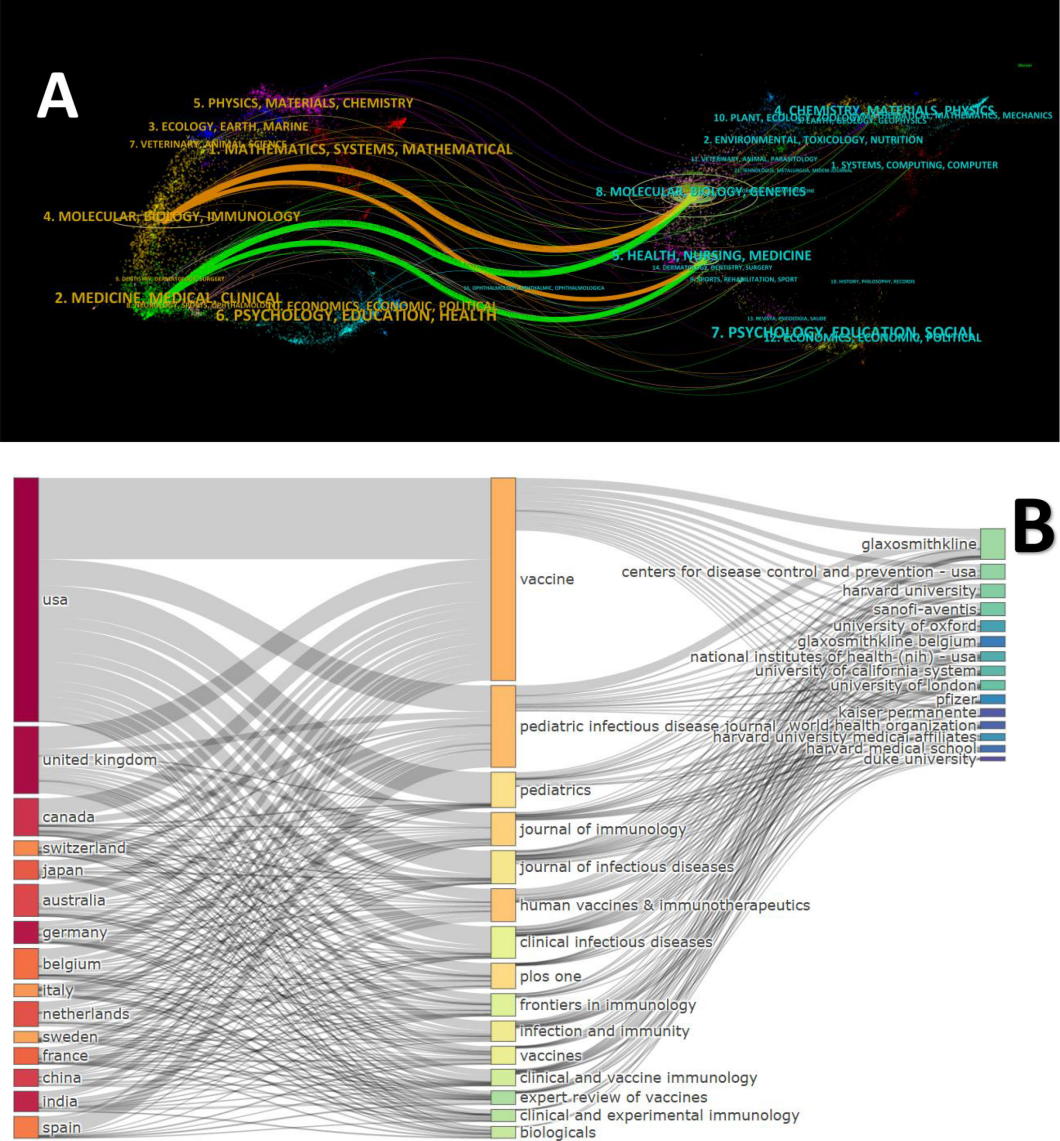
Interdisciplinary knowledge flow and structural relationships. **(A)** Dual-map overlay of journals: The colored paths show citation links from citing journals (left) to cited journals (right). **(B)** Three-fields plot: A Sankey diagram illustrating the connections between countries (left), journals (middle), and institutions (right).

Journal-based bibliometric analysis highlighted the field’s disciplinary distribution and inter-field citation relationships (**Table 4**, **Figure 5**). The journal Vaccine served as the primary platform for this field, accounting for nearly 10% of the total output, which underscores its role as the core repository for tetanus-related research. Interestingly, high-impact journals like Pediatrics and the Journal of Immunology showed significantly higher citation averages than the most prolific journals. This suggests that while specialized vaccine journals handle the bulk of field-specific reports, groundbreaking mechanistic and pediatric clinical studies are preferentially published in broader, high-impact medical journals (**Table 4**).

**Table 4:** Top 10 most productive journals in TT and TIG.

RRank	Journal	Publications	Citations	Citation per article	RIF(2024)	JCR(2024)
1	Vaccine	400	11381	28.45	3.5	Q2
2	The Pediatric Infectious Disease Journal	103	2264	21.98	2.2	Q3
3	Human Vaccines & Immunotherapeutics	75	1180	15.73	3.5	Q2
4	Journal of Immunology	72	9665	134.24	3.4	Q2
5	PLoS One	71	1456	20.51	2.6	Q2
6	Pediatrics	60	3851	64.18	6.4	Q1
7	The Journal of Infectious Diseases	50	1684	33.68	4.5	Q2
8	Infection and Immunity	47	2713	57.72	2.8	Q3
9	Vaccines	47	255	5.43	3.4	Q2
10	Clinical Infectious Diseases	40	2079	51.98	7.3	Q1

**Figure 5A** illustrates a dual-map overlay of journals, visualizing citation pathways from citing journals (left) to cited journals (right), with colored paths representing interdisciplinary flows. Dominant trajectories included connections from “Medicine, Medical, Clinical” and “Molecular, Biology, Immunology” on the left to “Health, Nursing, Medicine” and “Molecular, Biology, Genetics” on the right, indicating core knowledge transfer within biomedical domains. Overall, the disciplinary relationships exhibit a pattern of cross-field intersection followed by progressive integration. **Figure 5B** depicts a three-field plot (Sankey diagram) mapping interconnections among countries (left), journals (center), and institutions (right). The USA emerged as the primary contributor, channeling publications through Vaccine and Pediatrics to institutions like the Centers for Disease Control and Prevention (CDC) and Harvard University. The United Kingdom followed, with flows via Vaccine and The Pediatric Infectious Disease Journal to entities such as the University of Oxford and GlaxoSmithKline.

### 3.6 Reference citation burst analysis

**Figure 6:**
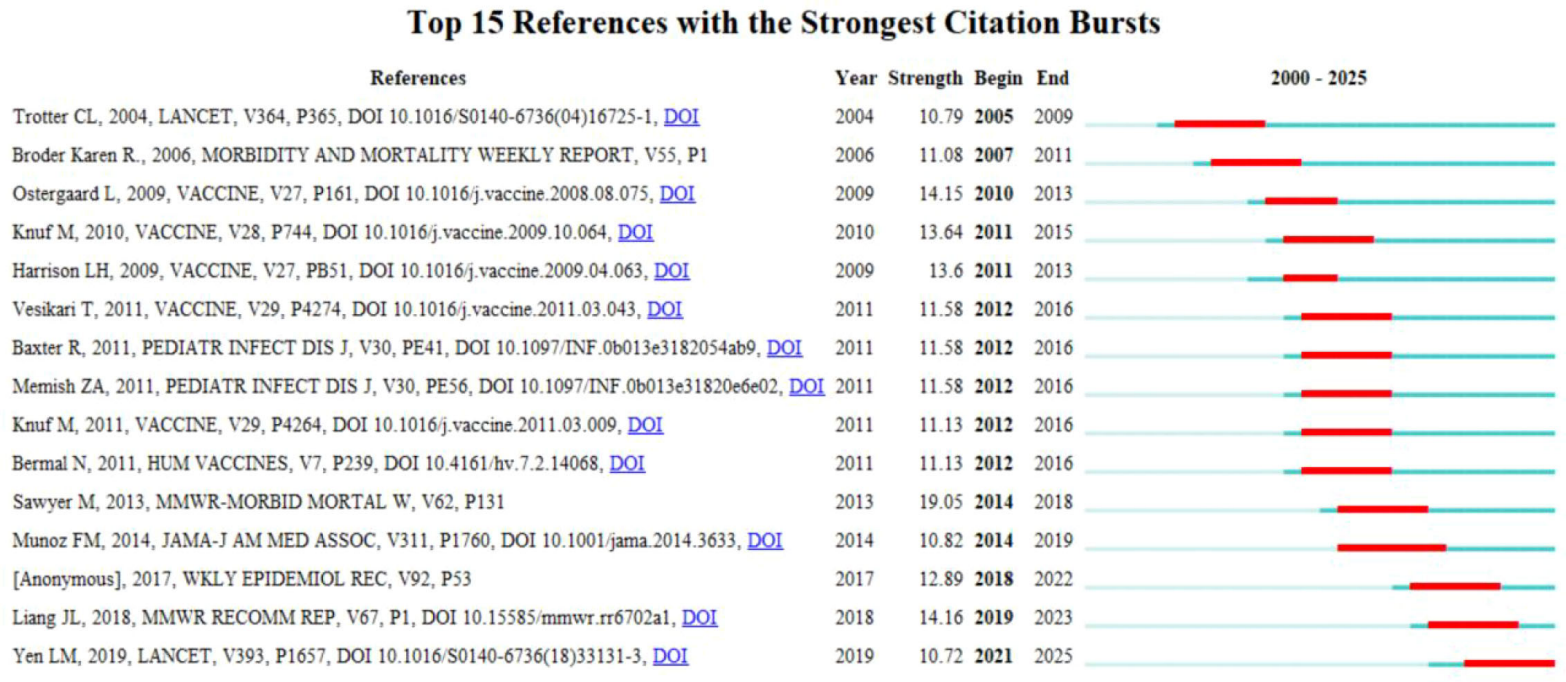
Top 15 references with the strongest citation bursts. The red segments indicate the duration and intensity of the citation bursts, highlighting key studies that significantly influenced the field during specific periods.

**Figure 6** displays the top 15 references with the strongest citation bursts in the field of TT and TIG from 2000 to 2025, reflecting key periods of heightened research attention. The strongest burst was observed for Sawyer et al (Sawyer et al., 2013), with a strength of 19.05 from 2014 to 2018, focusing on updated Advisory Committee on Immunization Practices (ACIP) recommendations for Tetanus Toxoid, Reduced Diphtheria Toxoid, and Acellular Pertussis Vaccine (Tdap) vaccination during pregnancy to enhance maternal and neonatal tetanus immunity. The report emphasized administering Tdap between 27 and 36 weeks gestation to maximize maternal antibody transfer to the fetus, particularly for women due for a tetanus booster (>10 years since last Td) or with incomplete tetanus vaccination histories, recommending a three-dose series (0, 4 weeks, 6–12 months) with Tdap replacing one dose to optimize protection against maternal and neonatal tetanus (Sawyer, 2013). This guidance has significantly influenced tetanus prevention strategies, addressing gaps in vaccination coverage and wound management during pregnancy. Another notable burst came from Munoz et al (Munoz et al., 2014), which had the longest burst period (2014 - 2019). This randomized trial confirmed the safety and immunogenicity of maternal Tdap vaccination, providing pivotal evidence for ACIP’s 2012 recommendation and influencing global neonatal tetanus prevention strategies (Munoz et al., 2014). In contrast, the Lancet Seminar by Yen and Thwaites (2019) has exhibited a sustained citation burst extending to the present, underscoring its comprehensive synthesis of tetanus epidemiology, prevention, and management, and highlighting its enduring relevance to global control strategies (Yen and Thwaites, 2019).

### 3.7 Keyword co-occurrence and trend analysis

**Figure 7:**
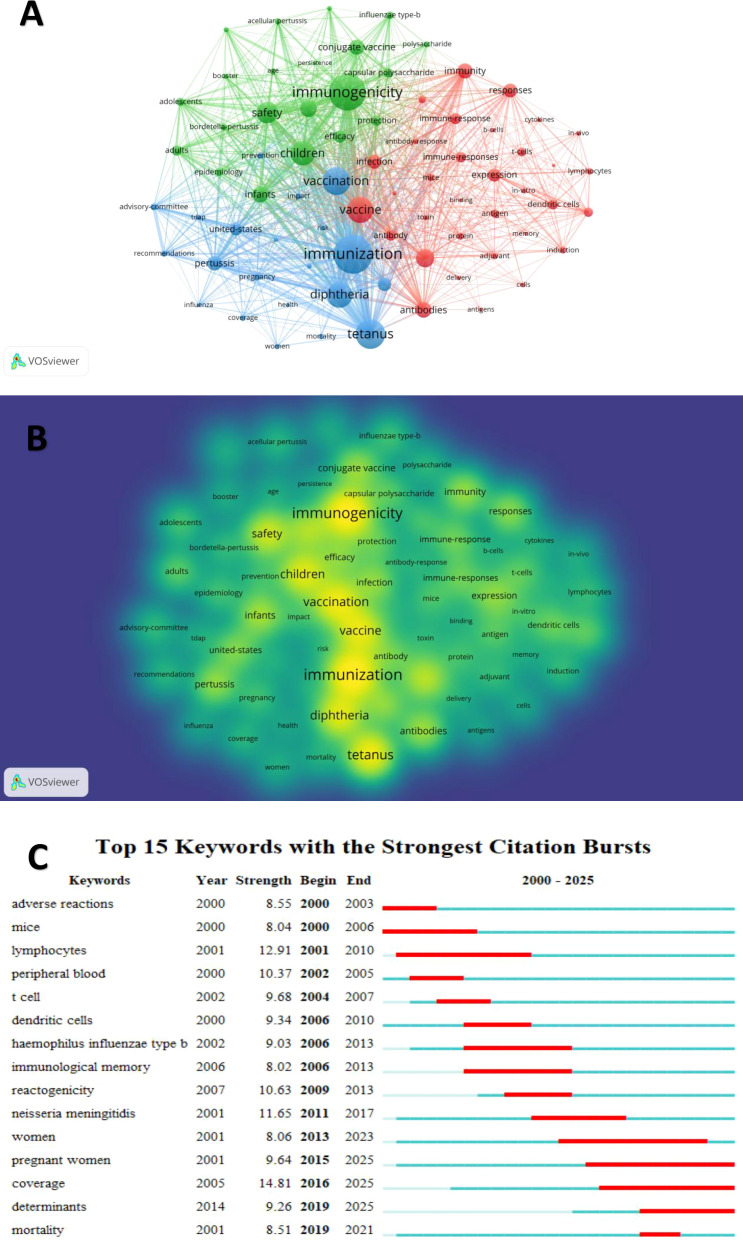
Keyword co-occurrence, density, and thematic evolution. **(A)** Co-occurrence map: Keywords are grouped into colored clusters representing major research themes. The thickness and number of lines between nodes represent the strength of relationship (co-occurrence) between keywords. **(B)** Density map: Higher color intensity reflects a higher frequency of keywords in core research areas. **(C)** Trend topics: The timeline illustrates the shift in research focus based on keyword emergence over time.

**Figure 7** illustrates keyword co-occurrence, density, and citation burst trends in research on TT and TIG. In the co-occurrence map (**Figure 7A**), keywords formed three primary clusters: a green cluster centered on clinical and epidemiological aspects (e.g., “immunogenicity,” “safety,” “efficacy,” “children,” “infants,” “protection”), a blue cluster emphasizing vaccination programs (e.g., “vaccination,” “immunization,” “diphtheria,” “pertussis,” “tetanus,” “coverage,” “pregnancy,” “recommendations”), and a red cluster focused on immunological mechanisms (e.g., “immune response,” “antibodies,” “antigen,” “cytokines,” “cells,” “t cells,” “b cells,” “mice”). Central nodes such as “vaccination,” “immunization,” “immunogenicity,” and “tetanus” exhibited high connectivity, underscoring their foundational role in the field, while peripheral terms like “*in vivo*,” “induction,” and “epidemiology” indicated specialized subthemes. The density map (**Figure 7B**) highlighted high-density regions around core keywords including “immunization,” “tetanus,” “safety,” “vaccine,” and “immunogenicity,” reflecting concentrated research activity in vaccine efficacy and immune protection. Lower-density areas encompassed emerging or niche topics such as “conjugate vaccine,” “capsular polysaccharide,” and “influenza vaccine type b,” suggesting potential avenues for future investigation. Citation burst analysis (**Figure 7C**) identified the top 15 keywords with the strongest bursts, revealing temporal shifts in research focus. The strongest burst was for “coverage” (strength 14.81, 2016–2025), indicating sustained emphasis on vaccination uptake and equity in tetanus prevention programs. While the second strongest burst occurred for “lymphocytes” (strength 12.91, 2001–2010), spanning 9 years and highlighting early interest in cellular immunity mechanisms. Ongoing bursts extending to 2025 included “pregnant women” (strength 9.64, 2015–2025, the longest burst), “coverage” (as noted), and “determinants” (strength 9.26, 2019–2025), pointing to contemporary priorities in maternal immunization strategies and factors influencing tetanus vaccine effectiveness in vulnerable populations. These patterns collectively demonstrate an evolution from basic immunological studies in the early 2000s to applied public health and policy-oriented research in recent years.

## 4 Discussion

### 4.1 General information

This bibliometric analysis delineates the evolving landscape of research on TT and TIG from 2000 to 2025, capturing a field marked by steady productivity and escalating citation impact, particularly in maternal and neonatal prevention strategies. Rather than indicating stagnation, this pattern suggests a gradual shift from disease-centered immunization toward broader platform optimization and policy integration within contemporary vaccinology. Annual publications, averaging 113.4 per year with a peak of 139 in 2004, reflect sustained global efforts to refine TT formulations and TIG applications amid declining but persistent disease burdens in LMICs (Dhir et al., 2021). The citation surge to 7,291 in 2021, may correlate with intensified focus on TT boosters during pregnancy, as evidenced by policy-driven research waves (Esposito et al., 2012). Incomplete 2025 data up to July 31 highlights the necessity for real-time tracking, especially as tetanus immunoglobulin shortages continue to challenge wound management protocols (Sudarshan et al., 2025). When benchmarked against the explosive growth seen in high-profile vaccine domains over the past two decades, tetanus research exhibits a “steady-state” productivity profile. This stability underscores its role as a mature scientific discipline that has transitioned from antigen discovery to the refinement of immunological platforms and the enhancement of multi-valent vaccine synergies.

Geographically, the USA’s preeminence with 1,130 publications underscores its leadership in tetanus vaccine trials and immunoglobulin safety evaluations (**Table 1**), a pattern echoed in other vaccine bibliometrics where resource-rich nations drive innovation (Ahmad et al., 2021a; Jones, 2024). England’s and Germany’s high citation averages (40.31 and 42.85, respectively) stem from seminal work on tetanus toxoid immunogenicity (Maple et al., 2000; Bartels et al., 2001; Bröker, 2016), while Switzerland’s exceptional average (88.41) arises from WHO-affiliated studies on global immunoglobulin distribution (World Health Organization, 2018). Emerging outputs from India (187 publications) and African nations like Ethiopia and South Africa signal vital LMIC contributions to neonatal tetanus elimination (**Figure 3**), aligning with WHO’s Maternal and Neonatal Tetanus Elimination (MNTE) initiative (Raza and Avan, 2019). Collaborative clusters, as mapped, facilitate cross-continental research efforts, yet also underscore persistent inequities: African intra-continental ties often focus on cost-effective toxoid delivery in endemic zones (Yaya et al., 2019; Sato and Fintan, 2020), while North American efforts emphasize advanced conjugate vaccines or frequently employ TT as a carrier protein (Zambrano et al., 2023). Although the Maternal and Neonatal Tetanus Elimination Initiative, launched in 1999 by the World Health Organization (WHO), the United Nations Children’s Fund (UNICEF) and the United Nations Population Fund (UNFPA), has eliminated maternal and neonatal tetanus from 47 of the 59 originally “high-risk” countries, 12 nations and territories remain vulnerable today (Thwaites et al., 2015; Etti et al., 2022). Our analysis reveals a critical mismatch: while these 12 remaining hotspots represent the highest clinical burden, they are seldom the primary focus of international collaborative networks, which predominantly circulate within HIC institutions. This “steady-state” productivity profile in the Global North may mask a stagnation in innovation tailored for low-resource settings—such as the development of heat-stable vaccine formulations or affordable recombinant antitoxins. To move beyond the current plateau, research efforts need shift from general immunological refinement toward targeted, field-applicable innovations within these vulnerable regions.

Institutionally, The U.S. Centers for Disease Control and Prevention (CDC), with 124 indexed publications and a mean citation rate of 41.15 (**Table 2**), has played a pivotal role in shaping global public health by issuing evidence-based guidelines on TT, reduced diphtheria toxoid, and acellular pertussis (Tdap) vaccination and codifying the indications and administration of tetanus immunoglobulin, thereby standardizing post-exposure prophylaxis worldwide (Liang, 2018). The University of Oxford has made significant contributions to tetanus-related vaccine research, particularly by highlighting the pivotal role of TT as a carrier protein-exemplified by the Vi-tetanus toxoid conjugate vaccine demonstrating strong immunogenicity and protection against typhoid fever (Jin et al., 2017)-and by advancing understanding of global tetanus prevention and management (Yen and Thwaites, 2019). Harvard University’s major contributions to tetanus vaccine research lie in public-health and long-term impact studies. Its scholars demonstrated through follow-up research on maternal TT immunization that the vaccine not only markedly reduces neonatal mortality but also promotes children’s educational and social development (Canning et al., 2011). While GSK has played a leading role in advancing tetanus vaccine research by developing and validating the combined Tdap vaccine Boostrix^®^, which demonstrated long-term immunogenicity, strong safety, and effective protection across adolescents, adults, and the elderly (Blatter et al., 2009; Van Damme et al., 2011; Weston et al., 2012; Vandermeulen et al., 2015). Network overlays (**Figure 4**) reveal temporal shifts: earlier blue nodes (circa 2008) from institutions like Harvard University and GSK emphasizing tetanus vaccine immunogenicity and safety profiles, while more recent yellow nodes (up to 2016) from entities such as the University of Oxford and CDC prioritize epidemiological analyses, vaccine development, and social promotion strategies for enhanced global adoption.

The distribution of funding sources (**Table 3**) provides a socio-economic explanation for the observed geographic disparities in research output. Our analysis reveals that tetanus research remains heavily reliant on a few high-income clusters, primarily the NIH in the USA and the MRC/Wellcome Trust in the UK. The recurrence of NIH-affiliated entries, such as NIAID and NCI, across more than a decade underscores a “sustained-investment” model that underpins the USA’s preeminence in the field. However, this concentration of capital highlights a significant research inequity: while the clinical burden of tetanus is most acute in LMICs, the financial power to dictate research agendas remains in the Global North. A pivotal shift was identified around 2010 with the emergence of the BMGF. By prioritizing global health equity, BMGF has bridged the “implementation gap” by funding large-scale maternal and neonatal tetanus elimination programs in the most vulnerable regions. This transition suggests that while government funding (e.g., NIH) drives foundational antigen and carrier-protein innovation, philanthropic investment is the primary engine for translational equity in endemic hotspots.

The journal-based bibliometric analysis indicates that Vaccine serves as the primary publication outlet for TT and TIG research, reflecting its central role in disseminating clinical and translational findings, while high-impact journals such as Journal of Immunology and Pediatrics contribute mechanistic and neonatal-focused insights. Dual-map overlay analysis highlights interdisciplinary knowledge flows from clinical medicine and molecular immunology toward public health and genetics, suggesting robust integration of basic, clinical, and applied research within the field. These patterns underscore the importance of targeted journals in shaping research visibility and guiding evidence-based vaccine policy.

### 4.2 Hotspots and frontiers

Recent bibliometric trends highlight maternal immunization as a dominant research hotspot. Keywords (**Figure 7C**) like “pregnant women”, “coverage”, and “determinants” exhibit strong citation bursts, reflecting sustained interest in vaccine uptake and equity. For example, maternal TT vaccination is now recognized as a critical strategy to cut neonatal mortality – one dose during pregnancy significantly lowers infant death rates (Naha et al., 2025). Global data underscore this impact: reported neonatal tetanus deaths fell by 85% from 2000 to 2018, and 47 of 59 high-risk countries have achieved elimination of maternal–neonatal tetanus (Dhir et al., 2021; Jones, 2024; Naha et al., 2025). Nevertheless, coverage gaps persist, especially in low-resource settings. These findings indicate a shift in research focus from vaccine efficacy toward implementation science. Strong citation bursts for “determinants” and “coverage” highlight a key policy transition: attaining the global tetanus elimination targets demands not only effective vaccines but also robust delivery systems that overcome socio-cultural barriers. Future public health policies should prioritize integrating tetanus vaccination into routine antenatal care packages, rather than maintaining standalone tetanus programs. Recent studies show that only under half of pregnant women in sub-Saharan Africa receive ≥2 doses of tetanus vaccine, and socio-economic factors (e.g. maternal/partner education, wealth, pregnancy intention) strongly influence uptake (Tamir et al., 2023). Frontiers in the LMICs area focus on tackling these barriers-for instance, integrating tetanus vaccination into routine antenatal visits has been linked to higher “protection at birth” rates (Giles et al., 2020), and addressing safety concerns and strengthening healthcare worker recommendations can improve maternal vaccine acceptance (Khan et al., 2024).

Beyond coverage issues, research into immunological underpinnings of tetanus vaccines remains a hotspot. Keyword analysis(**Figure 7C**) showed early interest evolving from cellular immunity explorations (e.g., “lymphocytes” burst, 2001–2010) to the assessments of antibody persistence and booster efficacy(e.g., “immunological memory” burst, 2006–2013). TT elicits robust humoral responses, with seroprotective antitoxin levels (>0.1 IU/ml) persisting for decades in vaccinated adults, supporting extended booster intervals beyond the conventional 10 years (Hammarlund et al., 2016). However, in individuals already protected against tetanus, booster immunization elicits an attenuated and short-lasting response. Those with pre-booster titers ≥1 IU/ml show less than a three-fold increase at one month and no meaningful improvement in protection, followed by a gradual two-fold decline over the subsequent six months (Danilova et al., 2005). Recent immunological analyses have further revealed that the long-term persistence of antitoxin antibodies is largely driven by long-lived plasma cells and memory B cells residing in the bone marrow, which can be rapidly reactivated upon antigen re-exposure (Hammarlund et al., 2017). From a clinical perspective, these immunological insights highlight a critical gap in emergency wound management. While antibody persistence is robust, our analysis and clinical experience suggest that many surgical trauma patients—particularly elderly individuals with cognitive decline or poor recall—are unable to provide accurate vaccination histories. This uncertainty often leads to either unnecessary over-immunization. Consequently, a promising research frontier lies in the development of point-of-care testing (POCT) devices, such as rapid tetanus antibody test strips. Such tools would enable clinicians to immediately determine a patient’s immune status upon injury, ensuring evidence-based administration of boosters or immunoglobulins, thereby optimizing emergency resource allocation and enhancing patient safety.

Complementing these findings, the keyword co-occurrence analysis (**Figure 7A**) revealed the frequent appearance of related terms such as diphtheria, pertussis, *Haemophilus influenzae* type b (Hib-TT), and conjugate vaccine, suggesting an expanding research interface between TT and other vaccine platforms. This trend indicates that the research scope has transcended the singular goal of tetanus prevention, evolving into a broader exploration of TT as a foundational carrier platform. These associations primarily reflect the dual role of TT-as a protective antigen in the DTaP (diphtheria–tetanus–pertussis) combination vaccine and as a carrier protein in several conjugate vaccines. TT’s well-established safety profile, strong T-cell immunogenicity, and ability to induce long-lasting immune memory make it a preferred carrier for polysaccharide-based vaccines such as Hib-TT, meningococcal (MenA-TT, MenC-TT), and typhoid Vi conjugate vaccines (Vi-TT), etc (Tejedor et al., 2008; Jin et al., 2017; Klein et al., 2017; Huang et al., 2023). The integration of TT into these leading vaccine domains underscores its enduring relevance in the wider field of vaccinology, where its immunological profile often dictates the success of multi-valent and conjugate systems. Recent studies have demonstrated that TT’s recombinant derivatives can enhance the immunogenicity of weakly immunogenic polysaccharide antigens and induce durable T-cell dependent antibody responses, primarily in preclinical or early-stage evaluations (Yu et al., 2020; Oldrini et al., 2023),. The frequent co-occurrence of these keywords highlights the evolving role of TT in modern vaccinology, extending beyond tetanus prevention toward broader applications as an immunogenic carrier molecule. This functional transition reflects the expanding role of tetanus toxoid in conjugate vaccinology. Tetanus toxoid (TT) has evolved from being merely a vaccine antigen to serving as a highly immunogenic carrier protein that facilitates T-cell dependent responses against polysaccharide antigens. Its frequent application in conjugate vaccine platforms underscores its established immunological profile. However, challenges such as carrier-induced epitopic suppression highlight ongoing technical frontiers in pediatric multivalent vaccine design.

Another emerging frontier is passive immunotherapy for tetanus. Global shortages of human tetanus immunoglobulin (HTIG) have long been a concern, and recent work has validated novel alternatives (Liang et al., 2025). Recent preclinical and translational studies have isolated highly potent human monoclonal antibodies (mAbs) and antibody fragments that neutralize tetanus neurotoxin (TeNT) by binding distinct functional epitopes, and several mAb combinations provide complete protection in animal models of tetanus (Minamitani et al., 2021; Wang et al., 2021; Manieri et al., 2022). Importantly, translational progress includes characterization of human mAb panels with *in vivo* neutralization and protective efficacy, and early clinical development programs and investigational products (including recombinant full-human mAbs and engineered fragments) are now reported, suggesting a potential pathway toward scalable, pathogen-free alternatives to polyclonal HTIG, pending further clinical evaluation (Liang et al., 2025). This emerging technological direction addresses a longstanding structural limitation in tetanus prophylaxis: the reliance on plasma-derived immunoglobulins. The growing research emphasis on monoclonal antibodies indicates increasing exploration of biotechnology-derived biologics with defined specificity and scalable production. Although such approaches have not yet totally replaced human tetanus immune globulin in clinical practice, they may offer strategic advantages in standardization and long-term supply sustainability, particularly in settings where plasma resources are limited.

As indicated by the keyword “delivery” in **Figure 7A**, advances in vaccine delivery systems represent another emerging research frontier in tetanus immunization, driven by goals of improving antigen stability, enhancing immunogenicity, simplifying administration, and enabling dose-sparing or single-visit schedules. Nanoparticle and microscale carriers (lipid/polymeric nanoparticles, microparticles, hydrogels) enable antigen protection, controlled release and targeted uptake by antigen-presenting cells, thereby potentiating both humoral and cellular responses to vaccines (Wang et al., 2023; Zhuo et al., 2024). TT-loaded microneedle patches have elicited immune responses comparable to intramuscular vaccination in animal models, offering painless and self-administrable delivery for improved vaccine accessibility (Arshad et al., 2023). Likewise, intranasal delivery of TT encapsulated in chitosan nanospheres and combined with cross-linked dextran microspheres elicited robust systemic IgG and mucosal sIgA responses in rabbits, underscoring the potential of nanoparticle-based nasal systems as innovative needle-free tetanus vaccination platforms (Pirouzmand et al., 2017). Collectively, these advances position advanced delivery platforms as a practical means to overcome logistical and immunological limitations of conventional tetanus vaccines and therefore constitute a high-priority area for translational research and clinical evaluation.

Taken together, these thematic evolutions highlight a key conceptual insight of this study: tetanus research is not characterized by explosive expansion, but by adaptive reinvention. While publication volumes remain relatively stable compared with emerging vaccine domains, innovation occurs through functional repurposing—transforming tetanus toxoid from a standalone antigen into a carrier platform, integrating maternal vaccination into broader antenatal systems, and exploring monoclonal antibody technologies to replace plasma-derived immunoglobulins. This pattern suggests that scientific vitality in mature vaccine fields may manifest not as rapid growth, but as platform diversification and translational integration. Recognizing this dynamic is essential for strategic funding allocation and future vaccine system design.

### 4.3 Limitations

This study has several limitations that should be acknowledged. First, as the literature search was limited to the WoSCC (SCI-EXPANDED) and English-language publications, relevant studies indexed in other databases (e.g., Scopus, PubMed, Embase) or published in non-English journals may have been overlooked, which may have led to underrepresentation of studies published in local journals from countries where tetanus remains endemic, particularly in low- and middle-income regions. However, the close concordance with a parallel PubMed search indicates robust coverage of the global core literature. Moreover, our analysis already captures significant LMIC contributions, including 187 publications from India and prominent collaboration networks involving Ethiopia, Kenya, and South Africa, aligning with ongoing Maternal and Neonatal Tetanus Elimination efforts in endemic areas. Second, bibliometric analyses inherently emphasize quantitative metrics such as publication and citation counts, which may not fully capture research quality, scientific innovation, or policy impact. Third, citation-based indicators are time-dependent, favoring older publications and potentially underrepresenting recent but high-quality studies, particularly those published after 2023. Furthermore, the partial inclusion of 2025 data may slightly influence interpretations of the most recent publication and citation trends, although this is a common practice in bibliometric analyses aiming for timely overviews. Despite these limitations, the present analysis provides a comprehensive and timely overview of global research trends on tetanus vaccines and immunoglobulins, offering valuable insights to guide future investigations and public health strategies.

## 5 Conclusion

This bibliometric analysis reveals that global research on tetanus vaccines and immunoglobulins has maintained steady growth with increasing interdisciplinary collaboration and technological innovation. More importantly, the findings demonstrate that tetanus research functions as a model for understanding how mature vaccine systems evolve within modern immunology. Rather than representing a static legacy field, tetanus serves as a translational bridge between classical toxoid immunization, conjugate vaccine engineering, maternal immunization policy frameworks, and next-generation biologics. While traditional focuses on immunogenicity and maternal–neonatal protection remain central, emerging trends such as recombinant antibodies, conjugate vaccine platforms using TT as a carrier, and novel delivery systems including microneedles and nanoparticles highlight a shift toward next-generation immunization strategies. These insights extend beyond tetanus itself, offering broader implications for sustaining innovation in other long-established vaccine platforms and for aligning scientific development with global health equity goals. Continued efforts integrating basic research, translational development, and equitable implementation will be critical to sustaining progress toward global tetanus elimination.

## Data Availability

The original contributions presented in the study are included in the article/**Supplementary Material**. Further inquiries can be directed to the corresponding author/s.

## References

[B1] Ahmad T. Haroon Khan M. Murad M. A. Baig M. Murtaza B. N. (2021a). Research trends in rabies vaccine in the last three decades: a bibliometric analysis of global perspective. Hum. Vaccin. İmmunother. 17, 3169–3177. 33945433 10.1080/21645515.2021.1910000 PMC8381806

[B2] Ahmad T. Murad M. A. Baig M. Hui J. (2021b). Research trends in COVID-19 vaccine: a bibliometric analysis. Hum. Vaccin Immunother. 17, 2367–2372. doi: 10.1080/21645515.2021.1886806. PMID: 33687303 PMC8475596

[B3] Arshad M. S. Gulfam S. Zafar S. Jalil N. A. Ahmad N. Qutachi O. . (2023). Engineering of tetanus toxoid-loaded polymeric microneedle patches. Drug Delivery Transl. Res. 13, 852–861. doi: 10.1007/s13346-022-01249-9. PMID: 36253518 PMC9576317

[B4] Bartels I. Jüngert J. Lugauer S. Stehr K. Heininger U. (2001). Immunogenicity and reactogenicity of a single dose of a diphtheria–tetanus–acellular pertussis component vaccine (DTaP) compared to a diphtheria–tetanus toxoid (Td) and a diphtheria toxoid vaccine (d) in adults. Vaccine 19, 3137–3145. doi: 10.1016/S0264-410X(01)00029-9. PMID: 11312009

[B5] Blatter M. Friedland L. R. Weston W. M. Li P. Howe B. (2009). Immunogenicity and safety of a tetanus toxoid, reduced diphtheria toxoid and three-component acellular pertussis vaccine in adults 19–64 years of age. Vaccine 27, 765–772. doi: 10.1016/j.vaccine.2008.11.028. PMID: 19041352

[B6] Bröker M. (2016). Potential protective immunogenicity of tetanus toxoid, diphtheria toxoid and Cross Reacting Material 197 (CRM197) when used as carrier proteins in glycoconjugates. Hum. Vaccin Immunother. 12, 664–667. doi: 10.1080/21645515.2015.1086048. PMID: 26327602 PMC4964734

[B7] Canning D. Razzaque A. Driessen J. Walker D. G. Streatfield P. K. Yunus M. (2011). The effect of maternal tetanus immunization on children's schooling attainment in Matlab, Bangladesh: Follow-up of a randomized trial. Soc Sci. Med. 72, 1429–1436. doi: 10.1016/j.socscimed.2011.02.043. PMID: 21507538

[B8] Danilova E. Shiryayev A. Kristoffersen E. K. Sjursen H. (2005). Attenuated immune response to tetanus toxoid in young healthy men protected against tetanus. Vaccine 23, 4980–4983. doi: 10.1016/j.vaccine.2005.05.028. PMID: 15985319

[B9] Deng D. S. Shen Y. F. Li W. J. Zeng N. Huang Y. F. Nie X. W. (2023). Challenges of hesitancy in human papillomavirus vaccination: Bibliometric and visual analysis. Int. J. Health Plann. Manage. 38, 1161–1183. doi: 10.1002/hpm.3665. PMID: 37309072

[B10] Dhir S. K. Dewan P. Gupta P. (2021). Maternal and neonatal tetanus elimination: where are we now? Res. Rep. Trop. Med. 12, 247–261. doi: 10.2147/RRTM.S201989. PMID: 34849046 PMC8627318

[B11] Esposito S. Bosis S. Morlacchi L. Baggi E. Sabatini C. Principi N. (2012). Can infants be protected by means of maternal vaccination? Clin. Microbiol. Infect. 18, 85–92. doi: 10.1111/j.1469-0691.2012.03936.x, PMID: 22862749

[B12] Etti M. Calvert A. Galiza E. Lim S. Khalil A. Le Doare K. . (2022). Maternal vaccination: a review of current evidence and recommendations. Am. J. Obstet. Gynecol. 226, 459–474. doi: 10.1016/j.ajog.2021.10.041, PMID: 34774821 PMC8582099

[B13] Ghalavand M. Saadati M. Ahmadi A. Abbasi E. Salimian J. (2018). Immunological evaluation of chitosan nanoparticles loaded with tetanus toxoid. Bratisl Lek Listy 119, 71–74. doi: 10.4149/BLL_2018_013. PMID: 29455539

[B14] Giles M. L. Mantel C. Muñoz F. M. Moran A. Roos N. Yusuf N. . (2020). Vaccine implementation factors affecting maternal tetanus immunization in low- and middle-income countries: Results of the Maternal Immunization and Antenatal Care Situational Analysis (MIACSA) project. Vaccine 38, 5268–5277. doi: 10.1016/j.vaccine.2020.05.084. PMID: 32586763 PMC7342017

[B15] Hammarlund E. Thomas A. Amanna I. J. Holden L. A. Slayden O. D. Park B. . (2017). Plasma cell survival in the absence of B cell memory. Nat. Commun. 8. doi: 10.1038/s41467-017-01901-w. PMID: 29176567 PMC5701209

[B16] Hammarlund E. Thomas A. Poore E. A. Amanna I. J. Rynko A. E. Mori M. . (2016). Durability of vaccine-induced immunity against tetanus and diphtheria toxins: A cross-sectional analysis. Clin. Infect. Dis. 62, 1111–1118. doi: 10.1093/cid/ciw066. PMID: 27060790 PMC4826453

[B17] Huang F. Jing X. B. Li Y. B. Wang Q. Liu S. L. Yang Z. R. . (2023). Optimization of the process for preparing bivalent polysaccharide conjugates to develop multivalent conjugate vaccines against Streptococcus pneumoniae or Neisseria meningitidis and comparison with the corresponding licensed vaccines in animal models. Curr. Med. Sci. 43, 22–34. doi: 10.1007/s11596-022-2652-y. PMID: 36680685 PMC9862236

[B18] Jin C. Gibani M. M. Moore M. Juel H. B. Jones E. Meiring J. . (2017). Efficacy and immunogenicity of a Vi-tetanus toxoid conjugate vaccine in the prevention of typhoid fever using a controlled human infection model of Salmonella Typhi: a randomised controlled, phase 2b trial. Lancet 390, 2472–2480. doi: 10.1016/S0140-6736(17)32149-9. PMID: 28965718 PMC5720597

[B19] Johns N. E. Cata-Preta B. O. Kirkby K. Arroyave L. Bergen N. Danovaro-Holliday M. C. . (2023). Inequalities in immunization against maternal and neonatal tetanus: A cross-sectional analysis of protection at birth coverage using household health survey data from 76 countries. Vaccines (Basel) 11. doi: 10.3390/vaccines11040752. PMID: 37112664 PMC10146835

[B20] Jones C. E. (2024). Progress toward achieving and sustaining maternal and neonatal tetanus elimination—worldwide, 2000–2022. Mmwr. Morbid. Mortal. Wkly. Rep. 73, 614–621. doi: 10.15585/mmwr.mm7328a1. PMID: 39024183 PMC11262825

[B21] Khan T. Malik S. Rafeekh L. Halder S. Desai S. Das Bhattacharya S. (2024). Facilitators and barriers to maternal immunization and strategies to improve uptake in low-income and lower-middle income countries: A systematic review. Hum. Vaccin Immunother. 20. doi: 10.1080/21645515.2024.2411823. PMID: 39473171 PMC11533802

[B22] Klein N. P. Abu-Elyazeed R. Cornish M. Leonardi M. L. Weiner L. B. Silas P. E. . (2017). Lot-to-lot consistency, safety and immunogenicity of 3 lots of Haemophilus influenzae type b conjugate vaccine: results from a phase III randomized, multicenter study in infants. Vaccine 35, 3564–3574. doi: 10.1016/j.vaccine.2017.05.018. PMID: 28536030

[B23] Lambo J. A. Nagulesapillai T. (2012). Neonatal tetanus elimination in Pakistan: progress and challenges. Int. J. Infect. Dis. 16, E833–E842. doi: 10.1016/j.ijid.2012.07.015. PMID: 22940280

[B24] Li J. Liu Z. C. Yu C. Tan K. W. Gui S. J. Zhang S. . (2023). Global epidemiology and burden of tetanus from 1990 to 2019: A systematic analysis for the Global Burden of Disease Study 2019. Int. J. Infect. Dis. 132, 118–126. doi: 10.1016/j.ijid.2023.04.402. PMID: 37086867

[B25] Liang J. L. (2018). Prevention of pertussis, tetanus, and diphtheria with vaccines in the United States: recommendations of the Advisory Committee on Immunization Practices (ACIP). Mmwr. Recommendations Rep. 67, 1–44. doi: 10.15585/mmwr.rr6702a1. PMID: 29702631 PMC5919600

[B26] Liang Z. J. Liu S. Guo W. Deng Z. Bin W. K. Yu A. Y. . (2025). Recombinant monoclonal antibody siltartoxatug versus plasma-derived human tetanus immunoglobulin for tetanus: a randomized, double-blind, active-controlled, phase 3 trial. Nat. Med. 31, 2673-81. doi: 10.1038/s41591-025-03791-8. PMID: 40628965 PMC12353795

[B27] Liu Y. Cheng Y. J. Yan Z. Ye X. T. (2018). Multilevel analysis of international scientific collaboration network in the influenza virus vaccine field: 2006-2013. Sustainability 10. doi: 10.3390/su10041232. PMID: 41725453

[B28] Liu Y. Q. Ye Y. Dai F. Bai L. Ji H. J. Su X. X. . (2025). Global research hotspots and trends in microglia in ischemic stroke. Front. Immunol. 16. doi: 10.3389/fimmu.2025.1622499. PMID: 41280893 PMC12635585

[B29] Loan H. T. Yen L. M. Kestelyn E. Hao N. V. Mai N. Thuy D. B. . (2018). A pilot study to assess safety and feasibility of intrathecal immunoglobulin for the treatment of adults with tetanus. Am. J. Trop. Med. Hyg. 99, 323–326. doi: 10.4269/ajtmh.18-0153. PMID: 29916342 PMC6090350

[B30] Manieri T. M. Takata D. Y. Targino R. C. Quintilio W. Batalha-Carvalho J. V. Da Silva C. . (2022). Characterization of neutralizing human anti-tetanus monoclonal antibodies produced by stable cell lines. Pharmaceutics 14. doi: 10.3390/pharmaceutics14101985. PMID: 36297421 PMC9611486

[B31] Maple P. Jones C. S. Wall E. C. Vyse A. Edmunds W. J. Andrews N. J. . (2000). Immunity to diphtheria and tetanus in England and Wales. Vaccine 19, 167–173. doi: 10.1016/S0264-410X(00)00184-5. PMID: 10930669

[B32] Minamitani T. Kiyose K. Otsubo R. Ito T. Akiba H. Furuta R. A. . (2021). Novel neutralizing human monoclonal antibodies against tetanus neurotoxin. Sci. Rep. 11. doi: 10.1038/s41598-021-91597-2. PMID: 34108521 PMC8190289

[B33] Munoz F. M. Bond N. H. Maccato M. Pinell P. Hammill H. A. Swamy G. K. . (2014). Safety and immunogenicity of tetanus diphtheria and acellular pertussis (Tdap) immunization during pregnancy in mothers and infants: A randomized clinical trial. Jamajama 311, 1760–1769. doi: 10.1001/jama.2014.3633. PMID: 24794369 PMC4333147

[B34] Naha S. K. Arpon M. Siddique R. T. Ripa F. R. Hasan M. N. Uddin M. J. (2025). A study of association between maternal tetanus toxoid immunization and neonatal mortality in the context of Bangladesh. PloS One 20. doi: 10.1371/journal.pone.0316939. PMID: 39823396 PMC11741588

[B35] Oldrini D. Di Benedetto R. Carducci M. De Simone D. Massai L. Alfini R. . (2023). Testing a recombinant form of tetanus toxoid as a carrier protein for glycoconjugate vaccines. Vaccines (Basel) 11. doi: 10.3390/vaccines11121770. PMID: 38140177 PMC10747096

[B36] Pirouzmand H. Khameneh B. Tafaghodi M. (2017). Immunoadjuvant potential of cross-linked dextran microspheres mixed with chitosan nanospheres encapsulated with tetanus toxoid. Pharm. Biol. 55, 212–217. doi: 10.1080/13880209.2016.1257032. PMID: 27927058 PMC6130596

[B37] Raza S. A. Avan B. I. (2019). Eliminating maternal and neonatal tetanus and promoting clean delivery practices through disposable clean birth kits. Front. Public Health 7. doi: 10.3389/fpubh.2019.00339. PMID: 31824909 PMC6886002

[B38] Sato R. Fintan B. (2020). Effect of cash incentives on tetanus toxoid vaccination among rural Nigerian women: a randomized controlled trial. Hum. Vaccin Immunother. 16, 1181–1188. doi: 10.1080/21645515.2019.1672493. PMID: 31567041 PMC7227726

[B39] Sawyer M. Liang J. L. Messonnier N. Clark T. A. (2013). Updated recommendations for use of tetanus toxoid, reduced diphtheria toxoid, and acellular pertussis vaccine (Tdap) in pregnant women—Advisory Committee on Immunization Practices (ACIP), 2012. Morbidity and Mortality Weekly Report, 62, 131–135. Available online at: https://www.cdc.gov/mmwr/preview/mmwrhtml/mm6207a4.htm 23425962 PMC4604886

[B40] Sudarshan R. Sayo A. R. Renner D. R. de Saram S. Godbole G. Warrell C. . (2025). Tetanus: recognition and management. Lancet Infect. Dis. 25, e645–e657. doi: 10.1016/S1473-3099(25)00292-0. PMID: 40543524 PMC7617864

[B41] Tamir T. T. Kassie A. T. Zegeye A. F. (2023). Prevalence and determinants of two or more doses of tetanus toxoid-containing vaccine immunization among pregnant women in sub-Saharan Africa: Evidence from recent demographic and health survey data. Vaccine 41, 7428–7434. doi: 10.1016/j.vaccine.2023.11.007. PMID: 37949753

[B42] Tejedor J. C. Moro M. Merino J. M. Gómez-Campderá J. A. García-del-Rio M. Jurado A. . (2008). Immunogenicity and reactogenicity of a booster dose of a novel combined Haemophilus influenzae type b-Neisseria meningitidis serogroup C-tetanus toxoid conjugate vaccine given to toddlers of 13–14 months of age with antibody persistence up to 31 months of age. Pediatr. Infect. Dis. J. 27, 579–588. doi: 10.1097/INF.0b013e31816b4561. PMID: 18536619

[B43] Thwaites C. L. Beeching N. J. Newton C. R. (2015). Maternal and neonatal tetanus. Lancet 385, 362–370. doi: 10.1016/S0140-6736(14)60236-1, PMID: 25149223 PMC5496662

[B44] Tosun S. Batirel A. Oluk A. I. Aksoy F. Puca E. Bénézit F. . (2017). Tetanus in adults: results of the multicenter ID-IRI study. Eur. J. Clin. Microbiol. Infect. Dis. 36, 1455–1462. doi: 10.1007/s10096-017-2954-3. PMID: 28353183

[B45] Uleanya N. D. (2018). Achieving neonatal tetanus elimination in Nigeria: undisclosed challenges and prospects. Trop. Doct 48, 25–30. doi: 10.1177/0049475516689538. PMID: 28147895

[B46] Van Damme P. McIntyre P. Grimprel E. Kuriyakose S. Jacquet J. M. Hardt K. . (2011). Immunogenicity of the reduced-antigen-content dTpa vaccine (Boostrix®) in adults 55 years of age and over: A sub-analysis of four trials. Vaccine 29, 5932–5939. doi: 10.1016/j.vaccine.2011.06.049. PMID: 21718738

[B47] Vandermeulen C. Theeten H. Rathi N. Kuriyakose S. Han H. H. Sokal E. . (2015). Decennial administration in young adults of a reduced-antigen content diphtheria, tetanus, acellular pertussis vaccine containing two different concentrations of aluminium. Vaccine 33, 3026–3034. doi: 10.1016/j.vaccine.2014.10.049. PMID: 25613716

[B48] Wang E. Y. Sarmadi M. Ying B. B. Jaklenec A. Langer R. (2023). Recent advances in nano- and micro-scale carrier systems for controlled delivery of vaccines. Biomaterials 303. doi: 10.1016/j.biomaterials.2023.122345. PMID: 37918182

[B49] Wang Y. M. Wu C. W. Yu J. F. Lin S. J. Liu T. Zan L. P. . (2021). Structural basis of tetanus toxin neutralization by native human monoclonal antibodies. Cell Rep. 35. doi: 10.1016/j.celrep.2021.109070. PMID: 33951441

[B50] Weston W. M. Friedland L. R. Wu X. F. Howe B. (2012). Vaccination of adults 65 years of age and older with tetanus toxoid, reduced diphtheria toxoid and acellular pertussis vaccine (Boostrix®): Results of two randomized trials. Vaccine 30, 1721–1728. doi: 10.1016/j.vaccine.2011.12.055. PMID: 22212127

[B51] World Health Organization . (2018). Tetanus vaccines: WHO position paper, February 2017-Recommendations. Vaccine 36, 3573–5. doi: 10.1016/j.vaccine.2017.02.034. PMID: 28427847

[B52] Yaya S. Kota K. Buh A. Bishwajit G. (2019). Antenatal visits are positively associated with uptake of tetanus toxoid and intermittent preventive treatment in pregnancy in Ivory Coast. BMC Public Health 19, 1467. doi: 10.1186/s12889-019-7847-1. PMID: 31694607 PMC6836543

[B53] Yen L. M. Thwaites C. L. (2019). Tetanus. Lancet 393, 1657–1668. doi: 10.1016/S0140-6736(18)33131-3. PMID: 30935736

[B54] Yu R. Xu J. J. Hu T. Chen W. (2020). The pneumococcal polysaccharide-tetanus toxin native C-fragment conjugate vaccine: The carrier effect and immunogenicity. Mediators Inflamm. 2020. doi: 10.1155/2020/9596129. PMID: 32714092 PMC7355367

[B55] Yusuf N. Raza A. A. Chang-Blanc D. Ahmed B. Hailegebriel T. Luce R. R. . (2021). Progress and barriers towards maternal and neonatal tetanus elimination in the remaining 12 countries: a systematic review. Lancet Glob. Health 9, E1610–E1617. doi: 10.1016/S2214-109X(21)00338-7. PMID: 34678200 PMC8551683

[B56] Zaman M. Chandrudu S. Toth I. (2013). Strategies for intranasal delivery of vaccines. Drug Delivery Transl. Res. 3, 100–109. doi: 10.1007/s13346-012-0085-z. PMID: 23316448 PMC3539070

[B57] Zambrano B. Peterson J. Deseda C. Julien K. Spiegel C. A. Seyler C. . (2023). Quadrivalent meningococcal tetanus toxoid-conjugate booster vaccination in adolescents and adults: phase III randomized study. Pediatr. Res. 94, 1035–1043. doi: 10.1038/s41390-023-02478-5. PMID: 36899125 PMC10000353

[B58] Zhou C. X. Yu G. R. Wang Q. L. (2025). Global research trends and hotspots in erysipelas: a bibliometric analysis from 2000 to 2024. Front. Med. (Lausanne) 12. doi: 10.3389/fmed.2025.1530278. PMID: 40400624 PMC12092343

[B59] Zhuo Y. L. Zeng H. X. Su C. Y. Lv Q. Z. Cheng T. Y. Lei L. J. (2024). Tailoring biomaterials for vaccine delivery. J. Nanobiotechnol. 22. doi: 10.1186/s12951-024-02758-0. PMID: 39135073 PMC11321069

