# Supplementary material for: Two decades of global research on tetanus vaccines and tetanus immunoglobulins: a comprehensive bibliometric analysis and integrative review (2000–2025)
Source: Front Cell Infect Microbiol. 2026 Mar 13;16:1766140. doi: 10.3389/fcimb.2026.1766140 (PMC13021605; doi:10.3389/fcimb.2026.1766140)
Supplement: Supplementary file 1 [file DataSheet1.docx]

Database: PubMed
Search formula: (tetanus vaccine*[Title/Abstract] OR tetanus toxoid*[Title/Abstract] OR tetanus immunoglobulin*[Title/Abstract] OR tetanus immune globulin*[Title/Abstract] OR tetanus antitoxin*[Title/Abstract])
Publication date: From January, 2000, to July, 2025

**Identification**

**Screening**

Records screened

(n =3922)

Records excluded other than articles or reviews

(n = 70)

3852 records identified

Articles(n =3571)
Reviews(n= 281)

Records excluded for being non-English

(n = 146 )

Records excluded for not related to the topic or with incomplete data (n = 656)

3706 records identified

English (n= 3706)

A total of 3050 PubMed records (2830 articles and 220 reviews) were retrieved for a feasibility-check comparison with the WoSCC dataset

**Analysis**

Supplementary Material 1
Flowchart of PubMed literature retrieval for database feasibility-check.
